# Efficacy and safety of sitagliptin treatment in older adults with moderately controlled type 2 diabetes: the STREAM study

**DOI:** 10.1038/s41598-022-27301-9

**Published:** 2023-01-04

**Authors:** Mototsugu Nagao, Jun Sasaki, Hitoshi Sugihara, Kyoko Tanimura-Inagaki, Taro Harada, Ichiro Sakuma, Shinichi Oikawa, STREAM Study Investigators, T. Asano, S. Aoyama, T. Fukushima, J. Yan, O. Hasegawa, K. Hosokawa, Y. Ishimaru, H. Kaito, R. Kanbara, K. Kanno, K. Kimura, S. Moritani, T. Okuda, M. Okuma, T. Okumura, H. Omuro, Y. Sawayama, H. Shuto, J. Tanaka, T. Tada, K. Tateoka, T. Terada, H. Tsuzuki, M. Yamada

**Affiliations:** 1 Department of Endocrinology, Metabolism and Nephrology, Graduate School of Medicine, Nippon Medical School, Tokyo, Japan; 2 International University of Health and Welfare, Fukuoka, Japan; 3 Caress Sapporo Hokko Memorial Clinic, Hokkaido, Japan; 4 Diabetes and Lifestyle-Related Disease Center, Fukujuji Hospital, Japan Anti-Tuberculosis Association (JATA), 3-1-24 Matsuyama, Kiyose, Tokyo 204-8522 Japan; 5 Nishimura Memorial Hospital, Tokyo, Japan; 6 Oomura Hospital, Tokyo, Japan; 7 Mitaka Health Care Clinic, Tokyo, Japan; 8 Kyowa Clinic, Tokyo, Japan; 9 Heisei Tateishi Hospital, Tokyo, Japan; 10 Hosokawa Internal Medicine Clinic, Tokyo, Japan; 11 Memorial Kumagaya Diabetes Clinic, Saitama, Japan; 12 Kaito Iin, Tokyo, Japan; 13 Kanbara Clinic, Tokyo, Japan; 14 Koiwa Station Clinic, Tokyo, Japan; 15 Hachijyo Hospital, Tokyo, Japan; 16 Moritani Clinic, Tokyo, Japan; 17 Okuda Clinic, Tokyo, Japan; 18 Yufuin Kosei Hospital, Oita, Japan; 19 Oshima Medical Center, Tokyo, Japan; 20 Omuro Clinic, Tokyo, Japan; 21 Fukuoka Red Cross Hospital, Fukuoka, Japan; 22 Minamikoshigaya-Kennshinkai Clinic, Saitama, Japan; 23 Kumanomae Clinic, Tokyo, Japan; 24 Sato Hospital, Tokyo, Japan; 25 Heart Clinic Minamisenjyu, Tokyo, Japan; 26 Aqua Medical Clinic, Tokyo, Japan; 27 Nakaichou Clinic, Tokyo, Japan; 28 Yamada Clinic, Okinawa, Japan

**Keywords:** Type 2 diabetes, Randomized controlled trials

## Abstract

Sitagliptin has been suggested as a treatment option for older adults with type 2 diabetes (T2D). However, no randomized controlled trial has been performed to evaluate the efficacy and safety of sitagliptin treatment in older Japanese patients with T2D. The STREAM study was a multicenter, open-label, randomized controlled trial. T2D outpatients aged 65–80 years with moderately controlled glycemic levels (HbA1c 7.4–10.4%) under lifestyle interventions without or with oral anti-diabetic drugs excluding DPP4 inhibitors or GLP-1 receptor agonists were recruited (n = 176). The participants were randomized into sitagliptin group (n = 88) who received sitagliptin as an initial or an additive anti-diabetic drug and control group (n = 88) who did not. The treatment goal was HbA1c level < 7.4%. Efficacy and safety during 12-month treatment period were investigated. The mean (± SD) ages were 70.6 ± 3.9 and 71.9 ± 4.4 years old in sitagliptin and control groups, respectively. According to a mixed-effects model analysis, average changes from baseline over the treatment period in fasting plasma glucose (FPG), HbA1c, and glycated albumin (GA) were − 27.2 mg/dL, − 0.61%, and − 2.39%, respectively, in sitagliptin group, and 0.50 mg/dL, − 0.29%, and − 0.93%, respectively, in control group. The reductions in FPG, HbA1c, and GA were significantly greater in sitagliptin group (P < 0.0001, P < 0.01, and P < 0.0001, respectively). There were no differences in the incidence of adverse effects, except for cystatin C elevation and platelet count reduction in sitagliptin group. Sitagliptin treatment effectively improved the glycemic profile without any serious adverse effects in older T2D patients.

**Trial registration number:** UMIN000010376.

## Introduction

The frequency of type 2 diabetes (T2D) in older ages has been reported to be increasing annually in Japan. According to a recent report from the Japan Ministry of Health, Labor and Welfare^1^, the prevalence rates of T2D were approximately 25% and 15% in men and women aged 60 years or older, respectively. Older adults with diabetes tend to have higher morbidity and mortality rates than those without diabetes^2^. In addition, they are at a high risk for polypharmacy, functional disabilities and common geriatric syndromes, such as cognitive impairment, depression, falls, and urinary incontinence^3^. In fact, both hyper- and hypoglycemia have been suggested to increase the risk of cognitive impairment and dementia^4–6^. In addition, in older adults with diabetes, a U-shaped relationship has been observed between glycemic level (HbA1c) and frailty incidence, where an HbA1c level of 7.6% was correlated with the lowest risk^7^. Therefore, less intensive glycemic control is recommended for older adults with T2D, as described in the guideline of the Joint Committee of the Japan Diabetes Society and the Japan Geriatrics Society on improving care in older adults with diabetes^8^.

In terms of diabetic macrovascular complications, a Japanese randomized trial, J-EDIT, which was designed to evaluate the benefits of a multifactorial intervention (including HbA1c levels < 6.9% in the treatment goal) in older adults with T2D failed to show a significant risk reduction in cardiovascular events or stroke by intensive treatment^9^. However, a J-shaped relationship was observed between stroke incidence and HbA1c in the J-EDIT study, where an HbA1c level of 7.3–7.9% was correlated with the lowest risk^10^. Furthermore, a recent analysis of a Japanese administrative medical database revealed that older diabetic patients with poor glycemic control were at a higher risk of cardiovascular events, but patients with HbA1c levels < 7.2% showed no further risk reduction by decreasing HbA1c^11^. Therefore, tight glycemic control should be avoided to prevent macrovascular complications in older adults with diabetes.

Dipeptidyl peptidase IV (DPP4) inhibitors are oral anti-hyperglycemic agents that enhance insulin secretion in a glucose-dependent manner, and are notably different from sulfonylureas^12^. They prolong the half-life of incretin hormones, such as glucagon-like peptide-1 (GLP-1) and glucose-dependent insulinotropic polypeptide (GIP), by inhibiting DPP4 activity. DPP4 inhibitors have been shown to decrease HbA1c levels by 0.55–0.88%^13^. Sitagliptin is a DPP4 inhibitor that has been consistently observed to lower HbA1c levels not only in monotherapy but also in combination therapies with metformin, pioglitazone, sulfonylurea, and insulin^14–18^. Furthermore, no serious adverse effects have been reported in previous studies, except for a slight increase in serum creatinine (Cr) levels^19,20^. In addition, among older adults with T2D, the efficacy and safety of sitagliptin have been suggested for both mono- and combination therapies^20–24^. Some single-arm observational studies have analyzed glycemic parameters and the incidence of hypoglycemia under sitagliptin monotherapy and combination therapy in older adults with T2D^20–22^. In addition, Terauchi et al.^24^ performed a randomized study (START-J trial) comparing sitagliptin monotherapy with sulfonylurea monotherapy in drug-naïve T2D patients aged ≥ 60 years. The START-J trial has shown non-inferiority on glycemic control and superior safety of sitagliptin monotherapy as compared to sulfonylurea monotherapy. However, to our knowledge, no randomized controlled trial has been performed to study the efficacy and safety of sitagliptin treatment in older adults with T2D under lifestyle interventions without or with oral anti-diabetic drugs in a real-world clinical setting.

Especially for Japanese patients with T2D who have persistent hyperglycemia despite treatment with lifestyle intervention, insulin secretagogues such as glinides or sulfonylureas have traditionally been selected as the initial anti-diabetic drug in the past decades because β-cell dysfunction, rather than insulin resistance, is the primary cause of hyperglycemia in these patients^25^. Therefore, we frequently encounter older adults with T2D who take glinides or sulfonylureas. For those patients with persistent hyperglycemia despite current treatments, including glinides/sulfonylureas, we consider sitagliptin could be an ideal candidate for add-on treatment. Therefore, to select sitagliptin treatment for older Japanese patients with T2D, it is necessary to have detailed drug information regarding the efficacy and safety of the drug, such as risk of hypoglycemia, influences on underlying comorbidities, and real-world clinical and laboratory adverse effects.

In the present study, we recruited moderately controlled Japanese T2D patients aged 65–80 years with ordinary physical activity, who had been already under diet and exercise interventions without or with oral anti-diabetic drugs excluding DPP4 inhibitors or GLP-1 receptor agonists. Then, the participants were randomly allocated into sitagliptin group who received sitagliptin as an initial or an additive anti-diabetic drug to current treatment regimens and control group who did not, to evaluate the efficacy and safety of sitagliptin treatment in older adults with T2D.

## Methods

### Study design

This study was designed as a multicenter, open-label, randomized controlled trial. This trial has been registered at the University Hospital Medical Information Network (UMIN) as the **S**itagliptin **TR**ial for Safety and Efficacy in **E**lderly P**A**tients with **M**oderately Controlled Type 2 Diabetes (STREAM) Study (UMIN000010376). Participant recruitment and follow-up were conducted in 25 hospitals and clinics across Japan from April 2012 to September 2015. The study conformed to the principles outlined in the Declaration of Helsinki and was approved by the Nippon Medical School Hospital Ethical Committee: study No. 223055, date of approval 2012-03-12. All participants provided written informed consent after receiving a plenary explanation of the study.

### Participants

Eligible patients were T2D outpatients with ordinary physical activity, aged 65–80 years with HbA1c levels of 7.4–10.4% (the percentage value of the National Glycohemoglobin Standardization Program) who were under non-pharmacological (diet and exercise) or pharmacological treatment with oral anti-diabetic drugs, and whose HbA1c levels were stable (within 0.1% in absolute value) in the past two months. Patients were ineligible if they had type 1 diabetes; were treated with DPP4 inhibitors, GLP-1 receptor agonists, or insulin; or if they had chronic renal failure (estimated glomerular filtration ratio [eGFR] < 30 mL/min/1.73 m^2^). Patients judged to be inappropriate to participate in the study were also excluded.

Eligible patients were anonymously reported to the registration office at the Department of Preventive Medicine, Kyushu University Graduate School of Medical Sciences (Fukuoka, Japan) and then randomly allocated to either sitagliptin treatment or not. Allocation was conducted according to the sequence of a randomization list, which was prepared a priori via the permuted block method with a block size of 10 containing equal assignments to the two arms. Patients with sitagliptin treatment (sitagliptin group) were administered sitagliptin 50 mg once daily. A reduced dose (sitagliptin 25 mg once a day) was administered to patients with an eGFR of 30–60 mL/min/1.73 m^2^. The treatment was continued for 12 months, and the treatment goal was an HbA1c level < 7.4%. The study physicians were requested not to change the anti-diabetic medication regimen during the first 3 months. On the other hand, in patients without sitagliptin treatment (control group), a new addition or dose increase of insulin secretagogues (glinides or sulfonylureas) was permitted if necessary. After the first 3 months, a new addition, dose change or cessation of oral anti-diabetic drugs excluding DPP4 inhibitors or GLP-1 receptor agonists were allowed for both groups. Antihypertensives, lipid-lowering drugs, and other concomitant mediations were requested not to change during the first 3 months for both groups.

### Outcomes

The primary efficacy outcomes were changes in glycemic parameters, including fasting plasma glucose (FPG), HbA1c, and glycated albumin (GA) levels, and the secondary outcomes were changes in body weight, body mass index (BMI), and insulin-related parameters (fasting insulin and proinsulin). Potential improvements (or suppression of deterioration) in nephropathy (serum Cr and cystatin C [CysC], Cr- and CysC-based eGFR, urinary albumin-Cr ratio [uACR]), retinopathy (ophthalmofundoscopic findings), and peripheral neuropathy (Achilles tendon reflex test) were also assessed as efficacy outcomes. Safety was assessed with respect to reported episodes of hypoglycemia, other adverse events, and laboratory measurements including hematology and serum liver enzyme levels.

### Measurements

Venous blood and urine samples were collected under fasting conditions at baseline and at 3, 6, and 12 months of treatment and were transferred to the central laboratory (SRL Inc., Tokyo, Japan). Body weight and blood pressure were measured at baseline and at every follow-up visit. Episodes of hypoglycemia or adverse events were collected by medical interviews at the follow-up visits. The findings of Achilles tendon reflex test (classified as normal, reduced, or disappeared) were recorded at baseline and at the 6- and 12-month visits. Ophthalmofundoscopic findings (classified as normal, simple retinopathy, pre-proliferative retinopathy, and proliferative retinopathy) were recorded at baseline and at the 12-month visit. Blood samples were centrifuged to separate the serum for laboratory measurements. The laboratory measurements for efficacy outcomes at 12 months were included only glucose metabolism parameters, serum Cr and CysC, and uACR simply for ease of conducting the study.

Plasma glucose levels were determined by the hexokinase UV method using Cicaliquid GLU L (Kanto Kagaku, Tokyo, Japan). HbA1c levels were determined by latex agglutination turbidimetry using RAPIDIA Auto HbA1c (Fujirebio Inc., Tokyo, Japan). GA levels were determined by the visible absorption spectrometry enzymatic method using Lucica GA-L (Asahi Kasei Pharma Co., Tokyo, Japan). Serum insulin and C-peptide levels were measured via chemiluminescent enzyme immunoassay commercial kits (Fujirebio, Inc.), and proinsulin levels were determined via a two-antibody radioimmunoassay (Denis Pharma K.K., Tokyo, Japan). Serum Cr was measured via the visible absorption spectrometry enzymatic method using Detaminer L CRE (Asahi Kasei Pharma Co.). CysC levels were measured using a colloidal gold immunoassay (Alfresa Pharma Co., Osaka, Japan). The eGFR was calculated based on age, sex, and serum Cr or CysC levels. The uACR was measured using an Autokit Micro Albumin (Fujifilm Wako Pure Chemical Co., Tokyo, Japan). Total cholesterol and triglycerides (TG) levels were determined using the cholesterol dehydrogenase UV method and an enzymatic method using Cholestest CHO and Pure Auto S TG-N (Sekisui Medical Co., Tokyo, Japan). High-density lipoprotein (HDL) cholesterol levels were measured via a direct assay using Cholestest N-HDL (Sekisui Medical Co.). Low-density lipoprotein (LDL) cholesterol levels were measured using the Friedewald formula. The non-HDL cholesterol level was calculated the subtracting HDL cholesterol level from the total cholesterol level. Serum aspartate aminotransferase (AST), alanine aminotransferase (ALT), gamma-glutamyl aminotransferase (GGT), and uric acid levels were determined by standard methods in an external laboratory. Insulin-related parameters and serum lipids were not measured at the 12-month follow-up visit. When the insulin level was > 30 μU/mL, insulin-related parameters, namely FPG and TG, were not included in the analyses because the blood samples were suspected to have been obtained in a non-fasting condition.

### Safety analysis

Clinical adverse events were recorded from participants’ medical records. All newly reported symptoms and diseases during the treatment period were defined as clinical adverse events. Adverse effects in laboratory measurements were defined in accordance with the Common Terminology Criteria for Adverse Effects (CTCAE) version 5^26^. However, adverse effect criteria are not always specified for follow-up measurements when the baseline values are outside the normal range. Therefore, in the present analysis, a value greater or less than the baseline value was defined as adverse effect.

### Sample size and statistical analysis

Based on our previous study in T2D patients treated with sitagliptin^27^, the target of enrollment in the present study was 200 patients. An efficacy outcome analysis was performed according to the per-protocol analysis (see below). The safety analysis was based on the patients who had been treated with and without sitagliptin and for whom safety measurements were available. Between-group comparisons were performed using Fisher’s exact test for proportions and unpaired t-test for means. Regarding the outcome measures of primary interest, a mixed-model repeated-measures analysis was used to assess temporal changes in the treatment groups. The difference from the baseline value was used as the dependent variable, and the mixed model included the treatment group, time (three time-points in the follow-up), and the time-by-group interaction as explanatory variables. An unstructured covariance matrix was used to model the correlations among repeated measures. Means by time and group and 95% confidence intervals (CIs) were estimated. We also carried out a between-group comparison by month for the sake of confirmation. Urinary albumin was highly skewed to the right side, and the values were transformed to natural logarithms in the comparison of the change from the baseline. Statistical significance was set at P < 0.05. All statistical analyses were performed using Stata Statistical Software Release 13 (StataCorp, College Station, TX).

## Results

### Patient characteristics

**Figure 1:**
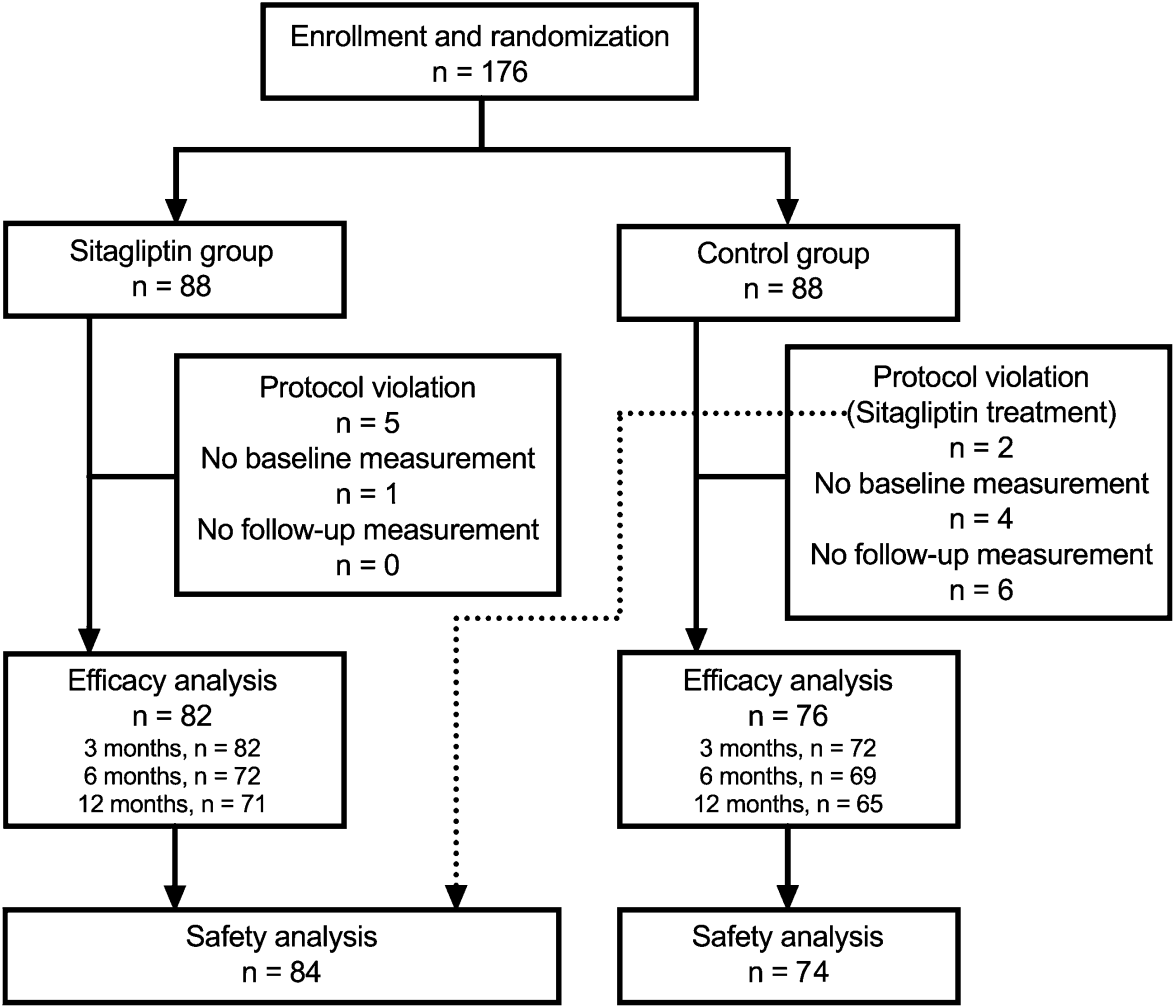
Flowchart of subject enrollment. A total of 176 patients were enrolled and randomly allocated into sitagliptin and control groups. Of the 176 participants, 18 were excluded from the efficacy analysis because of protocol violation, no baseline measurements, and no follow-up measurements. Two of the seven patients with protocol violation were treated with sitagliptin, despite being assigned to control group. These two patients who were treated with sitagliptin despite being assigned to control group were included in the safety analysis within sitagliptin group. The number of participants who completed blood tests of each follow-up visit is provided at the blocks of efficacy analysis.

A total of 176 patients were enrolled and randomly allocated into sitagliptin (n = 88) and control groups (n = 88). Males numbered 47 (57.3%) and 45 (59.2%) in sitagliptin and control group, respectively (P = 0.87). Of the participants, 18 were excluded from the efficacy analysis because of protocol violation (n = 7), no baseline measurements (n = 5), and no follow-up measurements (n = 6). Two of the seven patients with protocol violation were treated with sitagliptin, despite being assigned to control group. These two patients were therefore only included in the safety analysis. Finally, 158 patients (n = 82 in sitagliptin group and n = 76 in control group) were included in the efficacy analysis, as shown in Fig. 1. The baseline physical and laboratory measurements of the two groups are shown in Table 1. The mean age was ≥ 70 years in both sitagliptin and control groups. There were no significant differences in the laboratory measurements between the two groups. The comorbidities and medications at baseline are presented in Table 2. A higher frequency of diabetic retinopathy and a lower use of antidiabetics, namely sulfonylureas, were noted in sitagliptin group. Information about the use of antidiabetics other than sitagliptin during the treatment period is shown in Supplementary Table 1. In particular, the proportion of participants who took pioglitazone decreased from 26.0% at baseline to 19.4% at 12 months in sitagliptin group, and the proportion of those who took sulfonylureas increased from 52.1% at baseline to 61.8% at 12 months in control group. Detailed information about the changes of insulin secretagogues (sulfonylureas and glinides) are provided in Supplementary Tables 2 and 3.

**Table 1:** Physical and laboratory measurements at baseline.

Parameter	Sitagliptin		Control		P-value*
	n	Mean (SD)	n	Mean (SD)	
Age (year)	82	70.6 (3.9)	76	71.9 (4.4)	0.051
BMI (kg/m^2^)	69	24.9 (3.5)	63	24.7 (3.9)	0.73
SBP (mmHg)	77	131 (14)	71	132 (14)	0.73
DBP (mmHg)	77	73 (11)	71	70 (9)	0.08
FPG (mg/dL)	75	174 (54)	70	161 (53)	0.17
GA (%)	82	22.1 (3.8)	76	21.4 (3.0)	0.20
HbA1c (%)	82	7.8 (0.9)	76	7.6 (0.5)	0.08
Insulin (μU/mL)	75	9.6 (6.6)	70	8.1 (6.2)	0.16
Proinsulin (pmol/L)	74	33.9 (26.3)	69	28.5 (19.5)	0.17
Cr (mg/dL)	82	0.79 (0.21)	76	0.81 (0.23)	0.49
CysC (mg/L)	82	1.00 (0.23)	76	1.02 (0.23)	0.61
Cr-based eGFR	82	70 (17)	76	67 (16)	0.33
CysC-based eGFR	82	72 (18)	76	70 (15)	0.41
uACR (mg/g Cr)	82	176 (512)	76	114 (246)	0.33
T-C (mg/dL)	82	192 (43)	76	188 (37)	0.53
HDL-C (mg/dL)	82	55 (15)	76	54 (13)	0.79
LDL-C (mg/dL)	74	111 (35)	69	108 (34)	0.62
Non-HDL-C	82	138 (41)	76	134 (37)	0.58
Triglycerides (mg/dL)	75	141 (83)	70	142 (115)	0.98

*Between-group differences based on unpaired t-test. *BMI* body mass index, *Cr* creatinine, *CysC* cystatin C, *DBP* diastolic blood pressure, *eGFR* estimated glomerular filtration rate (mL/min/1.73 m^2^), *FPG* fasting plasma glucose, *GA* glycated albumin, *HDL-C* HDL cholesterol, *LDL-C* LDL cholesterol, *Non-HDL-C* non-HDL cholesterol, *SBP* systolic blood pressure, *T-C* total cholesterol, *uACR* urinary albumin-creatinine ratio.

**Table 2:** Comorbidity and medication at baseline.

Variable	Number of patients (%)		P-value *
	Sitagliptin (n = 77)	Control (n = 71)	
**Comorbidity**			
Retinopathy	7 (9.1)	0 (0.0)	0.01
Nephropathy	25 (32.5)	17 (23.9)	0.28
Neuropathy	4 (5.2)	4 (5.6)	1.00
CAD	11 (14.3)	9 (12.7)	0.81
Stroke	7 (9.1)	5 (7.0)	0.77
Hypertension	46 (59.7)	41 (57.7)	0.87
Dyslipidemia	51 (66.2)	47 (66.2)	1.00
Liver disease	10 (13.0)	6 (8.5)	0.43
Kidney disease	17 (22.1)	11 (15.5)	0.40
Other diseases	28 (36.4)	28 (39.4)	0.74
**Medication**			
Antidiabetics	53 (68.8)	60 (84.5)	0.03
Sulfonylureas	27 (35.1)	37 (52.1)	0.046
Metformin	36 (46.8)	33 (46.5)	1.00
Pioglitazone	20 (26.0)	17 (23.9)	0.85
α-Glucosidases	12 (15.6)	20 (28.2)	0.07
Glinides	1 (1.3)	2 (2.8)	0.61
Others^a^	2 (2.6)	1 (1.4)	1.00
Antihypertensives	43 (55.8)	35 (49.3)	0.51
Lipid-lowering drug	43 (55.8)	37 (52.1)	0.74
Other drugs	34 (44.2)	37 (52.1)	0.41

*Between-group differences based on Fisher’s exact test. *CAD* coronary artery disease. ^a^Epalrestat prescribed for diabetic neuropathy (n = 1 in sitagliptin group and n = 1 in control group) and imidapril for diabetic nephropathy (n = 1 in sitagliptin group) are listed as others (other antidiabetic medications).

### Efficacy analysis

**Figure 2:**
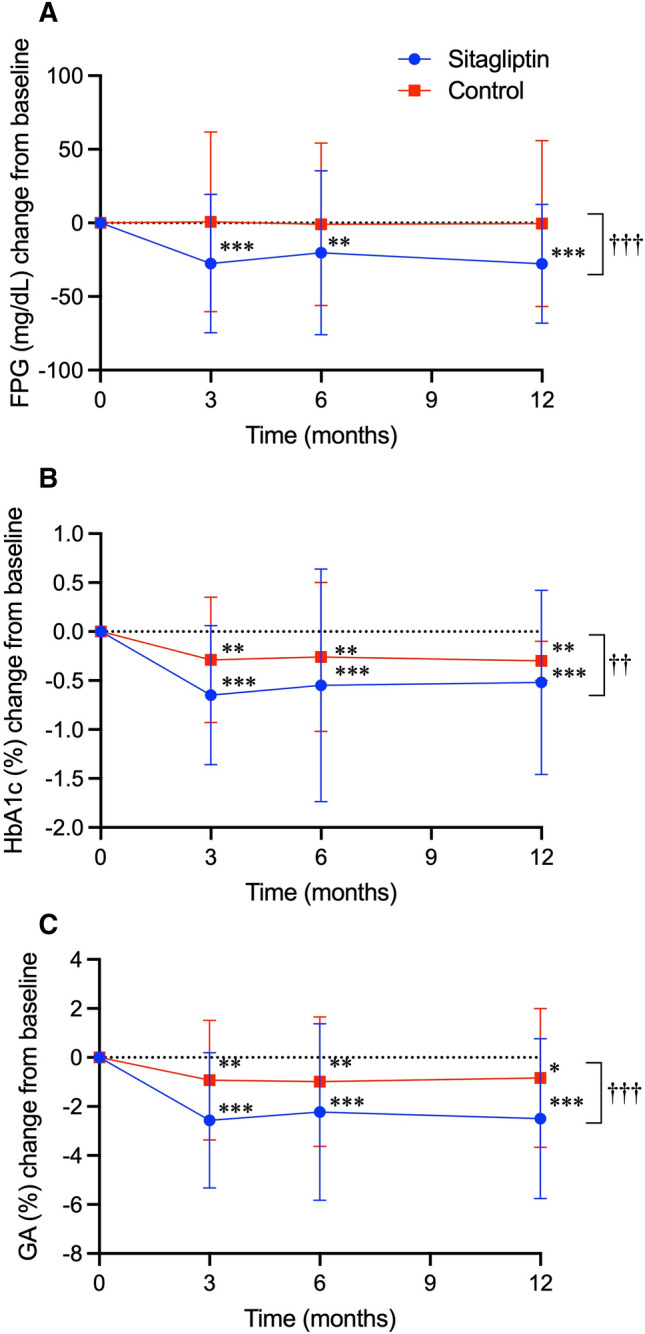
Treatment effect of sitagliptin on glycemic parameters. Changes in fasting plasma glucose (FPG, mg/dL) (**A**), glycated hemoglobin (HbA1c, %) (**B**) and glycated albumin (GA, %) (**C**) from baseline in sitagliptin and control groups are shown in closed circles with blue lines and closed squares with red lines, respectively. Data are expressed as mean ± standard deviation of the mean. *P < 0.05; **P < 0.01; ***P < 0.001 *vs*. baseline in each group on paired *t*-test. ^†^*P* < 0.05; ^††^*P* < 0.01; ^†††^*P* < 0.001 vs. control group on the mixed-effects model analysis.

The month-specific changes in glycemic parameters of primary outcomes are shown in Fig. 2. As compared to the baseline, FPG decreased significantly only in sitagliptin group, and HbA1c and GA decreased significantly in both groups at each follow-up month. Proportions of the participants who attained the therapeutic goal (HbA1c < 7.4%) in sitagliptin and control groups were 69.5% vs. 47.2% (P = 0.006), 69.4% vs. 52.2% (P = 0.04), and 64.8% vs. 53.8% (P = 0.22) at 3, 6, and 12 months, respectively. As shown in Table 3, the mixed model analysis revealed that the reductions in all the glycemic parameters over the treatment period were significantly greater in sitagliptin group than those in control group. The month-by-group interactions of the glycemic parameters were not significant. In the month-specific comparison in the mixed model analysis, the change in HbA1c showed a statistically significant between-group difference at 3 months (P < 0.001) and 6 months (P = 0.03), but not at 12 months (P = 0.10). As for GA, the between-group difference in the change was significant at 3 months (P < 0.001), 6 months (P < 0.01), and 12 months (P = 0.02).

**Table 3:** Average changes in parameters of primary and secondary outcomes during treatment based on a mixed-effects model analysis.

Parameter (unit)	Average change (95% CI)		P-value*	
	Sitagliptin	Control	Difference	Interaction
**Anthropometry**				
Body weight (kg)	0.06 (− 0.33; 0.45)	− 0.37 (− 0.78; 0.05)	0.14	0.46
BMI (kg/m^2^)	− 0.01 (− 0.17; 0.15)	− 0.18 (− 0.35; − 0.01)	0.14	0.59
**Glucose metabolism**				
FPG (mg/dL)	− 27.2 (− 37.7; − 16.8)	0.50 (− 10.3; 11.3)	< 10^−3^	0.81
GA (%)	− 2.39 (− 2.97; − 1.81)	− 0.93 (− 1.53; − 0.32)	< 10^−3^	0.65
HbA1c (%)	− 0.61 (− 0.77; − 0.45)	− 0.29 (− 0.46; − 0.12)	< 0.01	0.45
Insulin (μU/mL)^a^	− 0.98 (− 2.07; 0.11)	0.68 (− 0.44; 1.80)	0.04	0.49
Proinsulin (pmol/L)^a^	− 4.33 (− 7.84; − 0.82)	− 2.20 (− 5.79; 1.39)	0.43	0.10
**Renal function**				
Cr (mg/dL)	0.04 (0.03; 0.05)	− 0.01 (− 0.02; 0.01)	< 10^−4^	0.12
CysC (mg/L)	0.05 (0.03; 0.07)	0.00 (− 0.02; 0.02)	< 0.01	0.23
Cr-based eGFR	− 3.13 (− 4.30; − 1.96)	0.66 (− 0.55; 1.88)	< 10^−4^	0.10
CysC-based eGFR	− 3.89 (− 5.30; − 2.48)	0.17 (− 1.29; 1.63)	< 10^−4^	0.34
uACR (mg/g Cr)	− 19.6 (− 67.9; 28.6)	21.1 (− 29.1; 71.3)	0.27	0.39
ln[uACR]	− 0.21 (− 0.35; − 0.06)	0.06 (− 0.09; 0.21)	0.01	0.95

*‘Difference’ indicates the average change in parameters of primary and secondary outcomes caused by a specific treatment. ‘Interaction’ occurs when the length of treatment affected the average change in a selected parameter. ^a^Follow-up measurements were performed at 3 and 6 months. *BMI* body mass index, *Cr* creatinine, *CysC* Cystatin C, *eGFR* estimated glomerular filtration rate (mL/min/1.73 m^2^), *FPG* fasting plasma glucose, *GA* glycated albumin, *ln[uACR]* logarithmic urinary albumin-creatinine ratio, *uACR* urinary albumin-creatinine ratio.

Among the secondary outcomes shown in Table 3, changes in body weight and BMI did not differ between the two groups during treatment. Insulin levels were significantly reduced in sitagliptin group. Cr and CysC levels increased only in sitagliptin group; accordingly, reductions in Cr- and CysC-based eGFR were significantly greater in sitagliptin group. In contrast, a significantly greater reduction in logarithmic uACR was observed in sitagliptin group compared with that in control group. Patients with abnormal findings on ophthalmofundoscopy (n = 12) or the Achilles tendon reflex test (n = 19) at baseline did not show deterioration during the study period, and those without those findings did not develop any abnormalities.

### Safety analysis

Safety analyses were performed in 84 and 74 patients in sitagliptin and control groups, respectively (Fig. 1). The types of the adverse events are described in Table 4. Adverse events were recorded in eight (9.5%) and five (6.8%) patients in sitagliptin and control groups, respectively. These rates did not differ between the two groups. Laboratory adverse effects at specified time points during treatment are shown in Table 5. Cases with decreased platelet counts were noted only in sitagliptin group (n = 5), and there were no cases of grade 2 or higher. More cases of increased serum CysC levels were found at three and 12 months in sitagliptin group.

**Table 4:** Clinical adverse effects during treatment.

Adverse effect	Sitagliptin (n = 84)	Control (n = 74)
Hypoglycemia	1^a^	1
Vestibular vertigo	0	1^b^
Nasal bleeding	1	0
Acute gastroenteritis	1	0
Chronic atrophic gastritis	1^b^	0
Gastrointestinal symptom	1	0
Colon polyp	1^b^	0
Colon cancer	0	1
Alcoholic liver disorder	1	0
Acute myocardial infarction	0	1
Pruritus	1	0
Spinal canal stenosis	1	0
Scapulohumeral periarthritis	0	1
Drowning due to bicycle accident	0	1^b^

Adverse events were analyzed in all patients according to the actual use of sitagliptin. Clinical adverse events were assessed in 84 patients treated with sitagliptin and 74 patients treated without, for whom information on adverse effects was available. ^a^Two episodes were recorded. ^b^Recorded on different occasions by a single patient.

**Table 5:** Laboratory adverse effects by follow-up point.

Adverse event^a^	Month	Number (%)		P*	Cases of grade 2 or more
		Sitagliptin	Control		
WBC count increase	3	0/81 (0.0)	1/72 (1.4)	0.47	0^b^
	6	2/73 (2.7)	1/68 (1.5)	1.00	0^b^
	12	3/71 (4.2)	0/65 (0.0)	0.25	0^b^
WBC count decrease	3	2/81 (2.5)	2/72 (2.8)	1.00	0
	6	0/73 (0.0)	2/68 (2.9)	0.23	0
	12	2/71 (2.8)	6/65 (9.2)	0.15	C: 2
RBC count increase	3	1/81 (1.2)	2/72 (2.8)	0.60	NA
	6	1/73 (1.4)	1/68 (1.5)	1.00	NA
	12	2/71 (2.8)	0/65 (0.9)	0.50	NA
RBC count decrease	3	4/81 (4.9)	8/72 (11.1)	0.23	NA
	6	8/73 (11.0)	11/68 (16.2)	0.46	NA
	12	11/71 (15.5)	12/65 (18.5)	0.66	NA
Hemoglobin decrease	3	4/81 (4.9)	9/72 (12.5)	0.14	S: 1^c^
	6	8/73 (11.0)	13/68 (19.1)	0.24	S: 2^c^; C:1
	12	11/71 (15.5)	14/65 (21.5)	0.38	S: 2
Platelet count decrease	3	5/81 (6.2)	0/72 (0.0)	0.06	0
	6	5/73 (6.8)	0/68 (0.0)	0.06	0
	12	5/71 (7.0)	0/65 (0.0)	0.06	0
Serum AST increase	3	3/82 (3.7)	2/72 (2.8)	1.00	0
	6	6/73 (8.2)	2/69 (2.9)	0.28	S: 1
	12	8/71 (11.3)	2/65 (3.1)	0.10	0
Serum ALT increase	3	5/82 (6.1)	1/72 (1.4)	0.22	0
	6	6/73 (8.2)	2/69 (2.9)	0.28	0
	12	7/71 (9.9)	4/65 (6.2)	0.54	0
Serum GGT increase	3	1/82 (1.2)	5/72 (6.9)	0.10	C: 2^c^
	6	3/73 (4.1)	5/69 (7.2)	0.48	S: 1
	12	8/71 (11.3)	4/65 (6.2)	0.37	S: 1
Serum uric acid increase	3	9/82 (11.0)	4/72 (5.6)	0.26	0^b^
	6	6/73 (8.2)	4/69 (5.8)	0.75	0^b^
	12	7/71 (9.9)	3/65 (4.6)	0.33	0^b^
Serum Cr increase	3	15/82 (18.3)	10/72 (13.9)	0.52	0
	6	13/73 (17.8)	7/69 (10.1)	0.23	0
	12	14/71 (19.7)	8/65 (12.3)	0.26	0
Serum CysC increase	3	37/82 (45.1)	27/72 (37.5)	0.03	S: 1
	6	29/73 (39.7)	21/69 (30.4)	0.29	0
	12	44/71 (62.0)	20/65 (30.8)	< 10^−3^	S: 1
uACR increase	3	22/82 (26.8)	30/72 (41.7)	0.06	NA
	6	36/73 (49.3)	32/68 (47.1)	0.87	NA
	12	25/70 (35.7)	29/65 (44.6)	0.30	NA

*Between-group differences were based on Fisher’s exact test. ^a^Defined in accordance with the Common Terminology Criteria for Adverse Events (CTCAE) version 5. Adverse effects were not defined for RBC count or serum CysC levels in the CTCAE. An increase in serum CysC level was defined by applying the criteria used for serum creatinine. An increase (or decrease) in RBC count was defined when the value was greater (or less) than the upper (or lower) limit of the normal range and also greater (or less) than the baseline value. ^b^Grade 2 was not defined in the CTCAE, and no grade 3 cases were noted. ^c^Included one case each of grade. *AST* aspartate aminotransferase, *ALT* alanine aminotransferase, *C* control, *Cr* creatinine, *CysC* cystatin C, *GGT* gamma-glutamyl transferase, *NA* not applicable, *RBC* red blood cell, *S* sitagliptin, *uACR* urinary albumin-creatinine ratio, *WBC* white blood cell.

## Discussion

Here, we present the results of the STREAM study, a new randomized controlled trial that demonstrated the efficacy and safety of sitagliptin treatment in older T2D patients with moderate glycemic control. Improvements in the glycemic parameters were significantly greater in sitagliptin group than control group. In addition, the present study revealed that sitagliptin treatment improved albuminuria in older T2D patients. In the safety analyses, the incidence rate of adverse events did not differ between the two groups; however, sitagliptin treatment increased serum CysC levels and likely induced platelet count reduction. However, these laboratory adverse effects were not considered severe because there were no cases in which adverse effects developed to grade 3 as defined in the CTCAE.

In the present study, HbA1c levels decreased by 0.61% in sitagliptin group. The magnitude of HbA1c reduction by sitagliptin treatment was similar to that observed in a study on a combination therapy with sitagliptin and insulin^28^. In addition, Ujihara, et al.^22^ performed a single-arm observational study on the efficacy of sitagliptin add-on to non-insulin/GLP-1 pre-existing therapy in older adults with T2D. In the study, HbA1c levels decreased by 0.56–0.67% at 3 months after sitagliptin add-on to the other oral anti-diabetic drugs. Therefore, sitagliptin add-on is considered a good option to decrease HbA1c levels in patients already treated with any drug regimens without GLP-1-based drugs. In older subjects, GA has been reported to reflect current glycemic status more precisely than HbA1c because HbA1c can be affected not only by hematologic disorders such as iron deficiencies and chronic diseases, but also by age^29,30^. To our knowledge, this is the first study showing that sitagliptin treatment is effective in decreasing GA in older adults with T2D, as a profound reduction of GA by 2.39% was observed in sitagliptin group. GA has also been reported to reflect postprandial hyperglycemia and consequent glycemic fluctuation, both of which contribute to atherogenesis, more accurately than HbA1c in patients with T2D^31^. We and others have reported the usefulness of GA in predicting the presence of carotid atherosclerosis in patients with T2D^30,32^. Therefore, we believe that sitagliptin treatment may be beneficial in preventing diabetic macrovascular complications in older adults with T2D.

Notably, sitagliptin group showed a significant decrease in eGFR in association with significantly increased levels of Cr and CysC compared with control group. In contrast, a reduction in uACR was observed in sitagliptin group, suggesting that sitagliptin treatment may improve glomerular hyperfiltration. Such changes in renal function parameters after sitagliptin treatment are consistent with previous reports, including our own^27,33^. In fact, a previous study demonstrated that sitagliptin treatment decreased eGFR in older T2D patients with a high baseline eGFR, but not in those a low baseline eGFR^22^. Renal hemodynamic effects beyond glycemic control have been suggested for GLP-1-based therapies^34^. For instance, native GLP-1 infusion reduced the Cr clearance-measured GFR in obese subjects^35^. Additionally, liraglutide, a long-acting GLP-1 analog, reduced GFR and uACR in patients with T2D^36^. The renal effects of GLP-1 are partly explained by the inhibition of Na^+^–H^+^ exchanger 3 (NHE3), which assembles with DPP4 in the proximal brush border and reduces proximal sodium reabsorption and GFR through TGF activation^37^. However, other studies have not shown such renal effects in GLP-1-based therapies, including sitagliptin^38,39^. Therefore, further studies are needed to clarify the effects of DPP4 inhibitors, including sitagliptin, on renal function.

In the present study, adverse events were reported in both groups. Episodes of hypoglycemia were observed in one case in each group, but none of the patients developed severe hypoglycemia or dropped out due to hypoglycemia. In a randomized controlled study comparing treatment with sitagliptin and placebo, sitagliptin monotherapy did not cause hypoglycemia in 206 older T2D^40^. In an open-label observational study for older adults with T2D, there was no case of hypoglycemia after sitagliptin was added on to other oral anti-diabetic drugs, including sulfonylureas^22^. In the present study, we found that the incidence of hypoglycemia was not increased in sitagliptin group, which reinforces the evidence that sitagliptin is unlikely to cause severe hypoglycemic event in older adults with T2D.

In the laboratory adverse effect analysis, a decreased platelet count was noted in five cases in sitagliptin group, although there was no between-group difference. In fact, six cases of thrombocytopenia have been reported in patients treated with sitagliptin during post-marketing surveillance performed in Japan^41^. The five cases in the present study did not develop to grade 2 or higher defined in the CTCAE; therefore, the reduction in platelet counts was not severe. However, platelet counts should be carefully monitored in older adults treated with sitagliptin because the platelet count falls after 60 years of age^42^.

The present study had several limitations. First, the number of participants was relatively small. Second, older adults tend to have health problems, such as geriatric syndromes and impairments in physical and cognitive function, but we did not assess these disorders. Further studies stratified by the levels of cognitive function and activity of daily living are necessary to evaluate the risk and benefit of sitagliptin treatment in older adults with T2D. Third, the protocol did not refer to the changes in antihypertensives, lipid-lowering drugs, and other concomitant mediations. Therefore, we could not exclude a possibility that changes in these drugs might influence on renal function and also on glucose metabolism. Lastly, participants did not perform self-measurement of blood glucose (SMBG) or intermittently scanned continuous glucose monitoring (isCGM) during the treatment period. This means that occurrences of hypoglycemia unawareness and nocturnal hypoglycemia could not be ascertained in the present study.

In conclusion, the STREAM study revealed that sitagliptin treatment effectively improved the glycemic profile without any serious adverse effects, including self-reported hypoglycemia, in older T2D patients. Therefore, we consider sitagliptin as an ideal candidate to achieve better glycemic control in older adults with T2D who have persistent hyperglycemia under current lifestyle interventions and anti-diabetic drug regimens without GLP-1-based drugs.

## Supplementary Information

Supplementary Information.

## Data Availability

All data generated or analyzed during this study are included in this published article.
