# Supplementary material for: Efficacy and safety of sitagliptin treatment in older adults with moderately controlled type 2 diabetes: the STREAM study
Source: Sci Rep. 2023 Jan 4;13:134. doi: 10.1038/s41598-022-27301-9 (PMC9813363; doi:10.1038/s41598-022-27301-9)
Supplement: Supplementary file 1 — Supplementary Information. [file 41598_2022_27301_MOESM1_ESM.docx]

**Supplementary Table 1. Use of antidiabetics other than sitagliptin by follow-up point**

| Type of drug | Number of patients (%) | | P-value^*^ |
| --- | --- | --- | --- |
|  | Sitagliptin | Control |  |
| **Baseline** | (n = 77) | (n= 71) |  |
| Sulfonylureas | 27 (35.1) | 37 (52.1) | 0.046 |
| Metformin | 36 (46.8) | 33 (46.5) | 1.00 |
| Pioglitazone | 20 (26.0) | 17 (23.9) | 0.85 |
| α-Glucosidases | 12 (15.6) | 20 (28.2) | 0.07 |
| Glinides | 1 (1.3) | 2 (2.8) | 0.61 |
| Others^†^ | 2 (2.6) | 1 (1.4) | 1.00 |
| **3 months** | (n =77) | (n =71) |  |
| Sulfonylureas | 28 (36.4) | 45 (63.4) | 0.002 |
| Metformin | 35 (45.5) | 38 (53.5) | 0.41 |
| Pioglitazone | 14 (18.2) | 19 (26.8) | 0.24 |
| α-Glucosidases | 11 (14.3) | 20 (28.2) | 0.04 |
| Glinides | 1 (1.3) | 3 (4.2) | 0.35 |
| Others^†^ | 3 (3.9) | 1 (1.4) | 0.62 |
| **6 months** | (n =75) | (n =69) |  |
| Sulfonylureas | 28 (37.3) | 41 (59.4) | 0.01 |
| Metformin | 35 (46.7) | 37 (53.6) | 0.50 |
| Pioglitazone | 15 (20.0) | 20 (29.0) | 0.25 |
| α-Glucosidases | 11 (14.7) | 19 (27.5) | 0.07 |
| Glinides | 1 (1.3) | 3 (4.3) | 0.35 |
| Others^†^ | 2 (2.7) | 1 (1.4) | 1.00 |
| **12 months** | (n = 72) | (n = 68) |  |
| Sulfonylureas | 25 (34.7) | 42 (61.8) | 0.002 |
| Metformin | 35 (48.6) | 39 (57.4) | 0.31 |
| Pioglitazone | 14 (19.4) | 20 (29.4) | 0.24 |
| α-Glucosidases | 12 (16.7) | 18 (26.5) | 0.22 |
| Glinides | 1 (1.4) | 3 (4.4) | 0.36 |
| Others^†^ | 2 (2.8) | 1 (1.5) | 1.00 |

^*^, Based on Fisher’s exact test. ^†^, Including epalrestat and imidapril.

**Supplementary Table 2. Changes in use of sulfonylureas and glinides by follow-up point**

| Drug | Sitagliptin | | |  | Control | | |
| --- | --- | --- | --- | --- | --- | --- | --- |
|  | Ceased | Continued | Added |  | Ceased | Continued | Added |
| **3 months** |  |  |  |  |  |  |  |
| Glibenclamide | 0 | 3 | 0 |  | 0 | 6 | 0 |
| Gliclazide | 0 | 5 | 0 |  | 0 | 5 | 1 |
| Glimepiride | 0 | 19 | 1 |  | 2 | 24 | 9 |
| Mitiglinide | 0 | 1 | 0 |  | 0 | 2 | 1 |
| **6 months** |  |  |  |  |  |  |  |
| Glibenclamide | 0 | 3 | 0 |  | 1 | 4 | 0 |
| Gliclazide | 0 | 5 | 0 |  | 0 | 5 | 1 |
| Glimepiride | 0 | 18 | 2 |  | 2 | 23 | 8 |
| Mitiglinide | 0 | 1 | 0 |  | 0 | 1 | 1 |
| Repaglinide | 0 | 0 | 0 |  | 0 | 0 | 1 |
| **12 months** |  |  |  |  |  |  |  |
| Glibenclamide | 0 | 2 | 0 |  | 1 | 4 | 0 |
| Gliclazide | 0 | 5 | 0 |  | 0 | 5 | 1 |
| Glimepiride | 1 | 16 | 2 |  | 2 | 22 | 10 |
| Mitiglinide | 0 | 1 | 0 |  | 0 | 1 | 1 |
| Repaglinide | 0 | 0 | 0 |  | 0 | 0 | 1 |

Values are numbers of patients. Number of the patients (sitagliptin/control) were 77/71, 75/69, and 72/68 at 3, 6, and 12 months, respectively.

**Supplementary Table 3. Changes in dose of sulfonylureas and glinides of continuous use by follow-up point**

| Drug |  | Sitagliptin | | |  |  | Control | | |
| --- | --- | --- | --- | --- | --- | --- | --- | --- | --- |
|  | n | Decrease | No change | Increase |  | n | Decrease | No change | Increase |
| **3 months** |  |  |  |  |  |  |  |  |  |
| Glibenclamide | 3 | 0 | 3 | 0 |  | 6 | 1 | 4 | 1 |
| Gliclazide | 5 | 0 | 5 | 0 |  | 5 | 0 | 4 | 1 |
| Glimepiride | 19 | 3 | 16 | 0 |  | 24 | 1 | 23 | 0 |
| Mitiglinide | 1 | 0 | 1 | 0 |  | 2 | 0 | 2 | 0 |
| **6 months** |  |  |  |  |  |  |  |  |  |
| Glibenclamide | 3 | 0 | 3 | 0 |  | 4 | 0 | 4 | 0 |
| Gliclazide | 5 | 0 | 5 | 0 |  | 5 | 0 | 4 | 1 |
| Glimepiride | 18 | 3 | 15 | 0 |  | 23 | 2 | 20 | 1 |
| Mitiglinide | 1 | 0 | 1 | 0 |  | 1 | 0 | 1 | 0 |
| **12 months** |  |  |  |  |  |  |  |  |  |
| Glibenclamide | 2 | 0 | 2 | 0 |  | 4^*^ | 0 | 3 | 0 |
| Gliclazide | 5 | 0 | 5 | 0 |  | 5 | 0 | 4 | 1 |
| Glimepiride | 16 | 3 | 13 | 0 |  | 22 | 2 | 17 | 3 |
| Mitiglinide | 1 | 0 | 1 | 0 |  | 1 | 0 | 1 | 0 |

Values are numbers of patients. ^*^, Dose was unknown for one patient.
